# Characterization of the Antioxidant Effects of *γ*-Oryzanol: Involvement of the Nrf2 Pathway

**DOI:** 10.1155/2018/2987249

**Published:** 2018-03-14

**Authors:** W. Rungratanawanich, G. Abate, M. M. Serafini, M. Guarienti, M. Catanzaro, M. Marziano, M. Memo, C. Lanni, D. Uberti

**Affiliations:** ^1^Department of Molecular and Translational Medicine, University of Brescia, Brescia, Italy; ^2^Scuola Universitaria Superiore IUSS Pavia, Pavia, Italy; ^3^Department of Drug Sciences, University of Pavia, Pavia, Italy

## Abstract

*γ*-Oryzanol (ORY) is well known for its antioxidant potential. However, the mechanism by which ORY exerts its antioxidant effect is still unclear. In this paper, the antioxidant properties of ORY were investigated for its potential effects as a reactive oxygen and nitrogen species (ROS/RNS) scavenger and in activating antioxidant-promoting intracellular pathways utilizing the human embryonic kidney cells (HEK-293). The 24 h ORY exposure significantly prevented hydrogen peroxide- (H_2_O_2_-) induced ROS/RNS production at 3 h, and this effect was sustained for at least 24 h. ORY pretreatment also enhanced the activity of antioxidant enzymes: superoxide dismutase (SOD) and glutathione peroxidase (GPX). Interestingly, ORY induced the nuclear factor (erythroid-derived 2)-like 2 (Nrf2) nuclear translocation and upregulation of Nrf2-dependent defensive genes such as NAD(P)H quinone reductase (NQO1), heme oxygenase-1 (HO-1), and glutathione synthetase (GSS) at mRNA and protein levels in both basal condition and after H_2_O_2_ insult. Thus, this study suggested an intriguing effect of ORY in modulating the Nrf2 pathway, which is also involved in regulating longevity as well as age-related diseases.

## 1. Introduction

According to the Harmann theory of aging, oxidative stress is at the base of the mechanisms involved in aging processes [1] and contributes to the development of many age-related diseases including cancer, atherosclerosis, hypertension, diabetes, and neurodegenerative disorders [2–4]. Oxidative stress is defined as the imbalance of the production of free radicals, and the efficiency of antioxidant enzyme systems and the ability to activate antioxidant-promoting intracellular pathways. ROS/RNS are usually produced by living organisms as a result of normal cellular metabolism, but they can also be induced by different endogenous and exogenous insults [5]. Thus, during the life span, the organism is continuously at risk to be exposed to ROS/RNS beyond such a threshold level which the body tissues fail to counteract the damage. It is well known that ROS/RNS take part in physiological cell processes from low to moderate concentrations, but at high concentrations, they produce adverse modifications to the cellular macromolecules such as lipids, proteins, and DNA, affecting cell functions and survival [3, 6]. Antioxidant enzyme cascade such as superoxide dismutase (SOD), catalase (CAT), and glutathione peroxidase (GPx) acts as the first line of protection in counteracting ROS/RNS generation. These three enzymes work sequentially to neutralize free radicals. SOD catalyzes the dismutation of superoxide anion to H_2_O_2_, which is in turn neutralized to H_2_O by CAT or GPx [7]. In addition, antioxidant-promoting intracellular pathways contribute in maintaining redox steady state and in preventing ROS/RNS detrimental effects induced by stressors. The nuclear factor (erythroid-derived 2)-like 2 (Nrf2) transcription factor has emerged as a master regulator of cellular detoxification response and redox status since it protects the organism from pathologies caused or exacerbated by oxidative stress. A key role of the Nrf2 pathway has also emerged in the aging processes and in age-related diseases [8–10]. The Nrf2 pathway is an intrinsic mechanism to defense oxidative stress by inducing the transcription of up to 10% of human genes, which take part in different cellular functions such as ROS/RNS elimination, detoxification, xenobiotic metabolism, drug excretion, and nicotinamide adenine dinucleotide phosphate (NADPH) synthesis. Among the antioxidant pathways, the Nrf2 pathway promotes the transcription of NQO1, HO-1, and GSS because of the presence of an antioxidant responsive element (ARE) sequence in their promoter region [11, 12]. In basal condition, the activation of Nrf2 is mediated by disrupting its interaction and binding to Kelch-like ECH-associated protein 1 (Keap1), a cytosolic Nrf2 repressor, resulting in recruitment the Cul3 ubiquitin ligase to induce Nrf2 degradation via proteasome, thus acting as a sensor of oxidative stress [13].

In recent years, natural compounds have gained more attention since their therapeutic effects have been found important in the improvement of a lifestyle as well as in the protection against age-related diseases such as hyperlipidemia, inflammatory disorders, cardiovascular diseases, and cancer [14–16]. These observations might change the concept of food consumption and nutrition, not only for diminishing starvation and malnutrition but also for preventing morbidity and mortality of chronic diseases particularly related to free radical damage. Thus, dietary antioxidants containing antioxidative phytochemicals become crucial to counteract oxidative stress, consequently maintaining body homeostasis and redox balance [17].

Rice (*Oryza sativa* L.), a natural source of antioxidant, is rich in plenty of antioxidative components such as essential vitamin E complex, anthocyanins, and phenolic compounds [18]. As compared to other cereal grains, rice contains the highest special phenolic compound that is ferulic acid, which is presented in the form of a steryl ferulate called “*γ*-oryzanol.” ORY derived its name from rice because it was first discovered in rice bran oil and it is composed of a hydroxyl group [19–21]. ORY is a mixture of ferulic acid esters of phytosterols: triterpene alcohols and plant sterols [22]. The quantity of ORY and its compositions vary in the different types of rice. Some varieties of rice may exhibit an amount of ORY 8–10 times higher than vitamin E considered as one of the most potent natural antioxidants [23, 24].

The antioxidant properties of ORY have been investigated in *in vivo* and *in vitro* experiments, demonstrating its ability in preventing and reducing the ROS/RNS formation [16, 22]. Son et al. illustrated that mice subjected to a high-fat diet supplemented with ORY showed significantly lower amount of oxidative stress and lipid peroxidation when compared to mice fed with high-fat diet alone [25]. Similarly, in *Drosophila melanogaster*, ORY was found to ameliorate antioxidant activities, thereby preventing the oxidative damage [26].

However, how ORY exerts its antioxidant effects is still unclear. Here, the antioxidant mechanism of action of ORY was investigated by using HEK-293 cell line subjected to a subtoxic oxidative insult elicited by H_2_O_2_. This cellular model has been well recognized as a suitable tool to investigate the intracellular pathways involved in oxidative stress [27–29]. In particular, the mechanisms involved in ROS/RNS scavenging and the antioxidant-promoting intracellular pathway were characterized.

## 2. Material and Methods

### 2.1. Cell Culture and Treatments

Human embryonic kidney cells (HEK-293) were cultured in Dulbecco's modified Eagle's medium containing 10% fetal bovine serum, 2 mmol/L glutamine, 100 U/mL penicillin, and 100 *μ*g/mL streptomycin at 37°C in a 5% CO_2_-containing atmosphere. Oxidative insult was induced by adding 100 *μ*M of H_2_O_2_ to cells at 80% confluent monolayers for different times in accordance with the experimental paradigms.

### 2.2. Oryzanol and H_2_O_2_ Treatments

ORY was purchased from Sigma-Aldrich, Merck KGaA, Darmstadt, Germany. The powder was resuspended in ethanol at a concentration of 1 mg/mL. ORY was used at different concentration ranging from 1 to 20 *μ*g/mL in different period of time according with the experiments. H_2_O_2_ 30% *w/w* (Sigma-Aldrich, Merck KGaA, Darmstadt, Germany) was used to prepare an initial concentration of 1 mM that was then diluted in culture medium to obtain a final concentration of 100 *μ*M for different period of time as per the experiments.

### 2.3. Cell Viability

Cell viability was evaluated 24 h after the oxidative insult by MTT assay. Cells were incubated with 500 mg/mL of MTT (3-(4,5-dimethylthiazol-2-yl)-2,5-diphenyltetrazolium bromide) for 3 h at 37°C. Then, it was removed and cells were lysed with dimethyl sulfoxide. The absorbance at 595 nm was measured using a Bio-Rad 3350 microplate reader (Bio Rad Laboratories, Richmond, CA, USA). Cells treated with 0.2% Triton-X100 solution (Sigma-Aldrich, Merck KGaA, Darmstadt, Germany) were used to calculate the maximum toxicity. Data were expressed as percentage of cell viability over the corresponding controls.

### 2.4. Measurement of ROS/RNS Generation

Intracellular ROS levels were measured using the fluorescent dye 2′,7′-dichlorodihydrofluorescein diacetate (H_2_DCF-DA) (Thermo Fisher Scientific, Waltham, Massachusetts USA), a nonpolar compound that is converted into a nonfluorescent polar derivative (H_2_DCF) by cellular esterase after incorporation into cells. H_2_DCF is membrane permeable and is rapidly oxidized to the high fluorescent 2′,7′-DCF (DCF) in the presence of intracellular ROS. For the experiments, HEK-293 cells were pretreated with ORY_24h_ and then exposed to H_2_O_2_ oxidative insult at the different time points. At the end of the treatment, cells were washed in Hanks' balanced salt solution (HBSS) and then incubated in nitrogen-saturated HBSS with 20 mM H_2_DCF-DA dissolved in dimethyl sulfoxide (DMSO) for 30 minutes at 37°C 5% CO_2_. After washing twice in HBSS, intracellular DCF fluorescence, proportional to the amount of ROS/RNS, was evaluated by EnSight fluorescence 96-plate reader (EnSight™ Multimode Plate Reader, PerkinElmer) with excitation and emission wavelengths of 492 and 527 nm, respectively.

### 2.5. Subcellular Fractionation for Nrf2 Nuclear Translocation

Nuclear protein extracts were prepared by washing HEK-293 cells twice with ice-cold PBS. Cells were subsequently homogenized 15 times using a glass-glass dounce homogenizer in 0.32 M sucrose buffered with 20 mM Tris hydrochloride (Tris-HCl) (pH 7.4) containing 2 mM ethylenediaminetetraacetic acid (EDTA), 0.5 mM ethylene glycol-bis(2-aminoethylether)-N,N,N′,N′-tetraacetic acid (EGTA), 50 mM *β*-mercaptoethanol, and 20 *μ*g/mL leupeptin, aprotinin, and pepstatin. The homogenate was centrifuged at 300*g* for 5 minutes to obtain the nuclear fraction. An aliquot of the nuclear fraction was used for protein assay by the Bradford method, whereas the remaining was boiled for 5 min after dilution with sample buffer and subjected to polyacrylamide gel electrophoresis and immunoblotting as described below.

### 2.6. Western Blot Analysis

Total protein extracts were prepared by harvesting cells in 80 *μ*L of lysis buffer containing 50 mM Tris-HCl (pH 7.6), 150 mM sodium chloride (NaCl), 5 mM EDTA, 1 mM phenyl methyl sulphonyl fluoride, 0.5 mg/mL leupeptin, 5 mg/mL aprotinin, and 1 mg/mL pepstatin. Samples were sonicated and centrifuged at 15,000*g* for 30 minutes at 4°C. The resulting supernatants were isolated, and protein content was determined by a conventional method (BCA protein assay Kit, Pierce, Rockford, IL) before processing for Western blot analysis. Protein samples (30 *μ*g) of both total and nuclear extracts were electrophoresed in 10% or 12% acrylamide gel and electroblotted onto nitrocellulose membranes (Sigma-Aldrich, Merck KGaA, Darmstadt, Germany). Membranes were blocked for 1 h in 5% *w/v* bovine serum albumin in TBS-T (0.1 M Tris-HCl pH 7.4, 0.15 M NaCl, and 0.1% Tween 20) and incubated overnight at 4°C with primary antibodies. Primary antibodies were anti-Mn-superoxide dismutase (anti-SOD2, 1 : 200, Sigma-Aldrich, Merck KGaA, Darmstadt, Germany), anti-Cu-Zn superoxide dismutase (anti-SOD1, 1 : 300, Santa Cruz Biotechnology Inc., Heidelberg, Germany), anti-Nrf2 (1 : 2000, Novus, Bio-techne, Minneapolis, USA), anti-NQO1 (1 : 100, Novus, Bio-techne, Minneapolis, USA), anti HO-1 (1 : 2000, Novus, Bio-techne, Minneapolis, USA), anti-*β*-actin (1 : 1000, BD Biosciences, Franklin Lakes, NJ, USA), anti-lamin A/C (1 : 1000, BD Biosciences, Franklin Lakes, NJ, USA), and anti-*α* tubulin (1 : 1000, Sigma-Aldrich, Merck KGaA, Darmstadt, Germany). IRDye near-infrared dye-conjugated secondary antibodies (LI-COR, Lincoln, Nebraska, USA) were used. The immunodetection was performed using a dual-mode western imaging system Odyssey FC (LI-COR, Lincoln, Nebraska, USA). Quantification was performed using Image Studio Software (LI-COR, Lincoln, Nebraska, USA), and the results were normalized over the *α*-tubulin, *β*-actin, or lamin A/C signal.

### 2.7. Antioxidant Enzyme Activities

SOD activity was measured following the inhibition of epinephrine oxidation according to McCord's method [30]. Total protein extracts and epinephrine 0.1 M (Sigma-Aldrich, Merck KGaA, Darmstadt, Germany) were added to G buffer (0.05 M glycine and 0.1 M NaCl at pH 10.34), and the reaction was monitored measuring the decrease of absorbance at 480 nm. The activity of purified SOD enzyme (3000 U/mg, Sigma-Aldrich, Merck KGaA, Darmstadt, Germany) was also measured in each experiment as a positive control. Data were normalized for protein amount, and the results were expressed as U/mg using the molar extinction coefficient of 402 at 480 nm.

CAT activity was measured monitoring the decomposition of H_2_O_2_ according to Shangari and O'Brien [31]. In particular, total protein extracts were incubated in a substrate (65 *μ*M H_2_O_2_ in 6.0 mM PBS buffer pH 7.4) at 37°C for 60 s. The enzymatic reaction was stopped by the addition of 32.4 mM ammonium molybdate (Sigma-Aldrich, Merck KGaA, Darmstadt, Germany) and measured at 405 nm. The results were extrapolated by a standard curve (ranging from 12 U/mL to 0.25 U/mL) performed with purified CAT enzyme (20,100 U, Sigma-Aldrich, Merck KGaA, Darmstadt, Germany).

GPx activity was performed in accordance with Awasthi et al. [32], measuring NADPH oxidation at 340 nm in the reaction that involved oxidation of reduced glutathione (GSH) to glutathione (GSSG) followed by its reduction by glutathione reductase (GR). Total protein extracts were mixed with a reaction mix, containing 50 mM PBS with 0.4 mM EDTA at pH 7.0, 1.0 mM sodium azide solution, 1.0 mg *β*-NADPH, 100 U/mL GR, 200 mM GSH, and 10 *μ*L of 0.042% H_2_O_2_. The activity of purified GPx enzyme (116 U/mg) was also measured in each experiment as positive control. All the reagents were purchased from Sigma-Aldrich, Merck KGaA, Darmstadt, Germany. GPx activity was measured as nmol NADPH oxidized to NADP^+^/mg protein by using the molar extinction coefficient of 0.00622 at 340 nm, and the data were expressed as U/mg.

### 2.8. Quantitative Real-Time PCR

Total RNA was extracted from 5 × 10^6^ cells following TRIzol® reagent protocol (Invitrogen Corporation, Carlsbad, CA, USA). 2 *μ*g of total RNA was retrotranscribed with MMLV reverse transcriptase (Promega, Madison, Wisconsin, USA), using random hexamers in a final volume of 40 μL. Parallel reactions containing no reverse transcriptase were used as negative controls to confirm the removal of all genomic DNA. Nrf2, Keap1, NQO1, HO-1, and GAPDH primers were provided by Qiagen (Qiagen, Hilden, Germany). GAPDH was used as endogenous reference. Quantitative RT-PCR was performed with the ViiA7 Real-Time PCR System (Applied Biosystems, Foster City, CA, USA) using the iQ™SYBR Green Supermix method (Bio-Rad Laboratories, Richmond, CA, USA) according to manufacturer's instructions.

### 2.9. Statistical Analysis

The results are reported as mean ± standard error mean (SEM) or standard deviation (SD) of at least three independent experiments. Statistical differences were determined by the analysis of variance (one-way ANOVA) followed, when significant, by an appropriate post hoc test as indicated in figure legends. *p* value of ≤0.05 was considered statistically significant.

## 3. Results

### 3.1. Oryzanol Prevents H_2_O_2_-Induced ROS/RNS Generation and Cell Death

**Figure 1:**
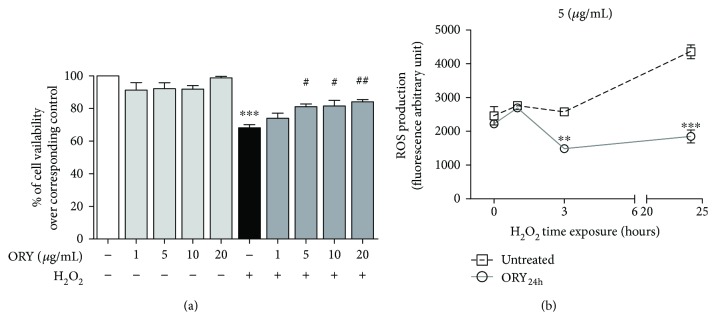
Oryzanol protects from cell death and decreases H_2_O_2_-induced ROS/RNS generation. (a) HEK-293 cells were pretreated for 24 h with ORY (ORY_24h_) at different concentrations and then stressed by the addition of 100 *μ*M H_2_O_2_ for 24 h. Cell viability was evaluated with MTT assay. Data are shown as percentage of cell viability compared with untreated cells; ^∗∗∗^*p* ≤ 0.001 versus untreated cells and ^##^*p* ≤ 0.01 and ^#^*p* ≤ 0.05 versus H_2_O_2_ group. (b) Effects of 5 *μ*g/mL ORY_24h_ on H_2_O_2_-induced ROS/RNS production were determined by H_2_DCF-DA oxidation using a fluorescence microplate reader. Fluorescence intensity of ORY_24h_ (open square symbol) after oxidative insult significantly decreased over time with ^∗∗^*p* ≤ 0.01 at 3 h and ^∗∗∗^*p* ≤ 0.001 at 24 h versus the corresponding untreated control group (open circle symbol). Bonferroni's multiple comparison test was used.

The antioxidant properties of ORY were evaluated in HEK-293 cells in baseline redox steady-state condition and after an acute oxidative insult. Cells were pretreated for 24 h with ORY (ORY_24h_) at different concentrations ranging from 1 to 20 *μ*g/mL followed by an acute oxidative insult elicited by 100 *μ*M H_2_O_2_. After 24 h, cell viability was evaluated with MTT assay. As shown in Figure 1(a), ORY_24h_ did not affect cell viability at any examined concentration. H_2_O_2_ alone reduced cell viability about 35% (*p* ≤ 0.001), but this mortality was rescued by ORY_24h_ at 5 *μ*g/mL (*p* ≤ 0.05) (Figure 1(a)). The minimum efficient concentration (5 *μ*g/mL) was used for the following experiments.

The effect of 5 *μ*g/mL ORY_24h_ on H_2_O_2_-induced ROS/RNS production was studied by monitoring the oxidation of the 2′-7′-dichlorofluorescein (DCF) probe from nonfluorescent-reduced state to be fluorescent in the presence of free radicals. 100 *μ*M H_2_O_2_ exposure initially induced a moderate production of ROS/RNS which significantly increased over time as assessed by accumulation of oxidized DCF (Figure 1(b), open square symbol). ORY_24h_ reverted H_2_O_2_-induced ROS/RNS generation at 3 h, and this effect was sustained at least until 24 h (Figure 1(b), open circle symbol).

### 3.2. Oryzanol Modulates the Antioxidant Enzyme Activities

**Figure 2:**
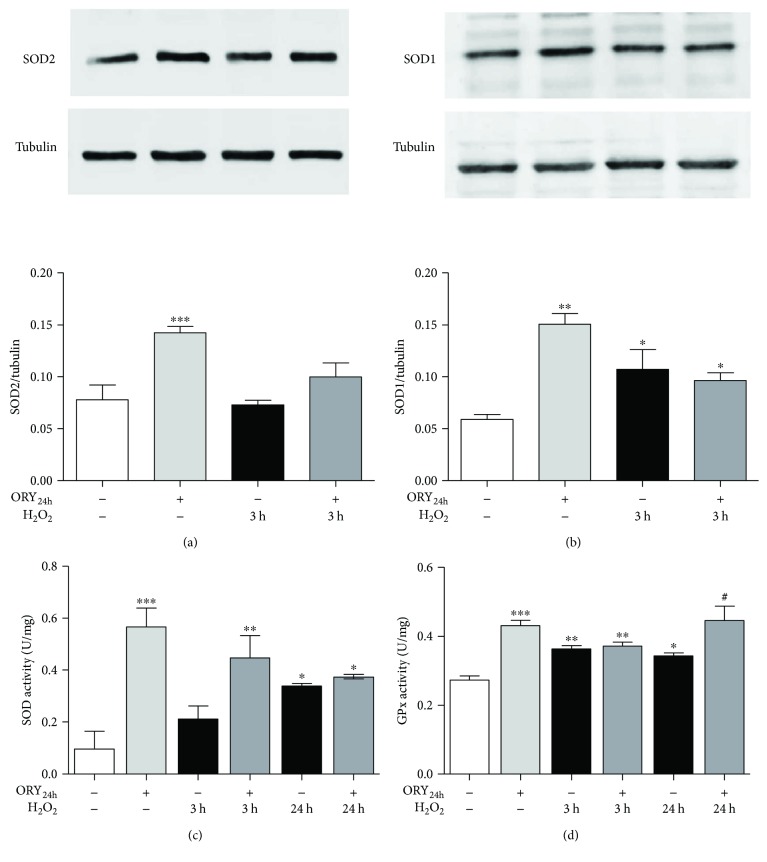
Oryzanol effect on antioxidant enzyme activities and MnSOD and Cu-ZnSOD expression. HEK-293 cells were pretreated with 5 *μ*g/mL ORY for 24 h followed by 100 *μ*M H_2_O_2_ for 3 h or 24 h. (a, b) Effect of ORY_24h_ on the expression of MnSOD (SOD2) and Cu-ZnSOD (SOD1) in H_2_O_2_-induced oxidative stress. The protein expression was assessed by Western blotting. Tubulin expression was used as loading control. Data are presented as mean ± SEM; ^∗∗∗^*p* ≤ 0.001 and ^∗∗^*p* ≤ 0.01 versus untreated cells. The activity of total SOD (c) and GPx (d) enzymes was assessed, as reported in the Material and Methods, before and after 3 h and 24 h of H_2_O_2_ oxidative insult. The results are represented as mean ± SEM; ^∗∗∗^*p* ≤ 0.001, ^∗∗^*p* ≤ 0.01, and ^∗^*p* ≤ 0.05 versus untreated cells and ^#^*p* < 0.05 versus the corresponding H_2_O_2_ control group. Bonferroni's multiple comparison test was used.

Different studies suggested that ORY could exert its antioxidant effects by modulating the endogenous antioxidant enzyme activities. In particular, SOD has been found to be positively regulated by ORY [26, 33, 34]. Here, we evaluated the protein expression and activity of SOD enzymes in the presence of ORY_24h_ alone or followed by oxidative insult. H_2_O_2_ insult did not affect the protein expression of the mitochondrial MnSOD (SOD2, Figure 2(a)) but significantly increased the cytosolic Cu-Zn SOD protein levels (SOD1, Figure 2(b)) that also reflected an increase of SOD activity induced by the oxidative insult (Figure 2(c)). ORY_24h_ alone significantly enhanced the protein expression of both SOD2 (mean ± SEM: untreated cells 0.078 ± 0.014 versus ORY_24h_ 0.143 ± 0.006; *p* ≤ 0.001) and SOD1 (mean ± SEM: untreated cells 0.059 ± 0.005 versus ORY_24h_ 0.150 ± 0.011; *p* ≤ 0.01) (Figures 2(a) and 2(b)) as well as the total SOD activity (mean ± SEM: untreated cells 0.098 ± 0.066 versus ORY_24h_ 0.566 ± 0.072; *p* < 0.001Figure 2(c)). In SOD1 protein expression, the combination of ORY_24h_ followed by H_2_O_2_ significantly maintained higher compared to untreated cells (mean ± SEM: 0.097 ± 0.007; *p* ≤ 0.05), but no difference was highlighted compared to H_2_O_2_ alone (mean ± SEM: 0.107 ± 0.019). Interestingly, in the presence of an acute oxidative insult (3 h of H_2_O_2_), ORY_24h_ maintained higher total SOD activity compared to H_2_O_2_ alone (mean ± SEM: ORY_24h_ + H_2_O_2_ 0.4448 ± 0.085 versus H_2_O_2_ 0.214 ± 0.048 Figure 2(c)). We also evaluated the activity of CAT and GPx, two enzymes that work downstream of SOD. No differences in terms of CAT activity were found compared to the different treatments (data not shown). Differently, GPx activity was found to be modulated by ORY. ORY_24h_ alone increased GPx activity (mean ± SEM: 0.43 ± 0.016) compared to untreated one (mean ± SEM: 0.27 ± 0.011; *p* ≤ 0.001Figure 2(d)). H_2_O_2_ acute insults at 3 h also induced an increase in GPx activity that was confirmed by the ORY pretreatment without further affecting the activity levels. A long lasting oxidative insult of H_2_O_2_ at 24 h which showed ORY_24h_ pretreatment still maintained higher GPx activity suggesting an active alarm system to detoxify from radicals. Altogether, these data confirmed the antioxidant effects of ORY through the modulation of endogenous antioxidant enzyme activities.

### 3.3. Oryzanol Activates Nrf2 Pathway

Activation of the Keap1/Nrf2 pathway and the consequent induction of its antioxidant genes trigger an elaborate network of protective mechanisms against oxidative damage [35]. When exposed to an oxidative insult, Keap1 undergoes conformational changes thereby disrupting its binding to Nrf2, which in turn promotes the Nrf2 translocation into the nucleus and activates the transcription-mediated protective responses. [36].

Thus, ORY was investigated to verify whether it might affect the Nrf2 pathway. We focused on the activation of Nrf2 signaling by analyzing its translocation into the nucleus and its ability to induce NQO1 and HO-1, the two prototypical cytoprotective Nrf2-target genes related to cellular stress response.

**Figure 3:**
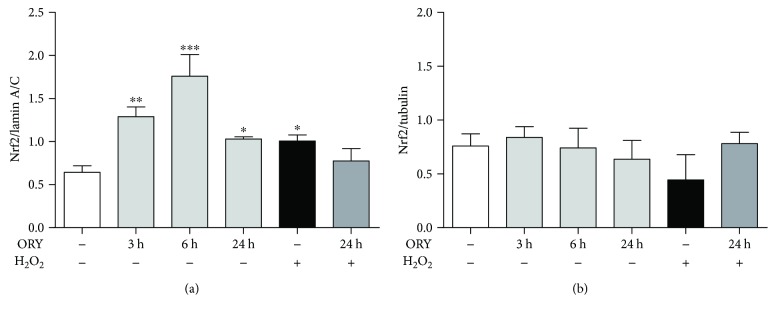
Nrf2 nuclear translocation induced by oryzanol. HEK-293 cells were pretreated with ORY for 3, 6, and 24 h and then stressed with 100 *μ*M H_2_O_2_ for 3 h. Nuclear and cytoplasmic fractions were isolated as described in the Material and Methods. (a) Nuclear expression of Nrf2 was assessed by Western blotting and lamin A/C expression was used as loading control. Data are represented as mean ± SD; ^∗∗∗^*p* ≤ 0.001, ^∗∗^*p* ≤ 0.01, and ^∗^*p* ≤ 0.05 versus untreated cells, Dunnett's multiple comparison test. (b) Cytoplasmic expression of Nrf2 was assessed by Western blotting, and tubulin expression was used as loading control. Data are represented as mean ± SD.

In basal condition, 3 h exposure of ORY significantly increased Nrf2 nuclear expression which was further enhanced at 6 h and decreased at 24 h in comparison with untreated cells (mean ± SEM: ORY_3h_ 1.28 ± 0.11; ORY_6h_ 1.76 ± 0.25; ORY_24h_ 1.03 ± 0.02 versus untreated 0.64 ± 0.07; *p* ≤ 0.01, *p* ≤ 0.001, and *p* ≤ 0.05, respectively, Figure 3(a)). As a positive control, 100 *μ*M H_2_O_2_ at 3 h treatment increased higher Nrf2 nuclear levels than those found in untreated cells (mean 1.01 ± 0.07; *p* ≤ 0.05) and ORY_24h_ (Figure 3(a)). The pretreatment of ORY_24h_ followed by 3 h H_2_O_2_ did not significantly differ in the extent of activation from the ORY_24h_ or H_2_O_2_ alone. To further sustain these results, we also investigated the Nrf2 expression in the cytosolic fraction. At 3, 6, and 24 h, ORY did not modify the Nrf2 cytoplasmatic expression, which expressed as ratio Nrf2/tubulin (Figure 3(b)). Likewise, a similar trend was observed in the presence of H_2_O_2_ alone or with ORY (Figure 3(b)).

**Figure 4:**
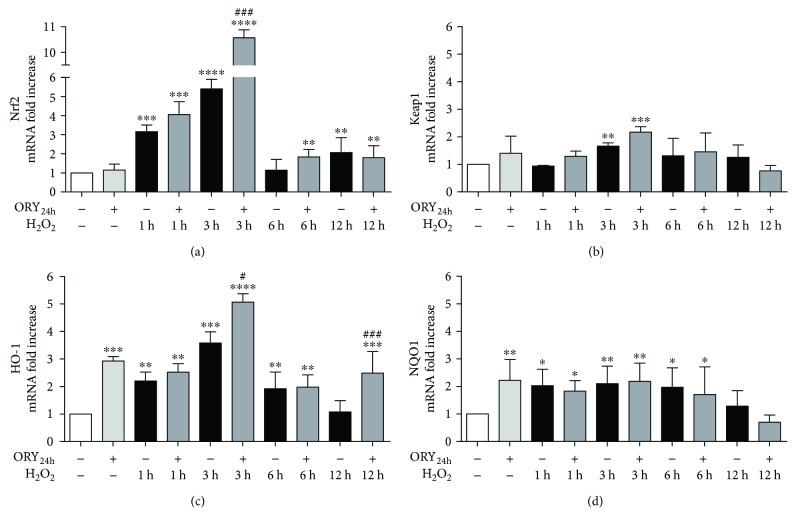
Oryzanol activates the transcription of Nrf2 target genes. HEK-293 cells were pretreated with 5 *μ*g/mL ORY for 24 h followed by a time course of 100 *μ*M H_2_O_2_ (1, 3, 6, and 12 h). Cells were then processed for measuring Nrf2 (a), Keap1 (b), HO-1 (c), and NQO1 (d) mRNA levels by real-time PCR. GAPDH was used to normalize the results. Data are shown as mean ± SEM. Statistically significant differences were represented as follows: ^∗∗∗∗^*p* ≤ 0.0001, ^∗∗∗^*p* ≤ 0.001, ^∗∗^*p* ≤ 0.01, and ^∗^*p* ≤ 0.05 versus untreated cells and ^###^*p* ≤ 0.001 and ^#^*p* ≤ 0.05 versus the corresponding H_2_O_2_ control group, Bonferroni's multiple comparison test.

**Figure 5:**
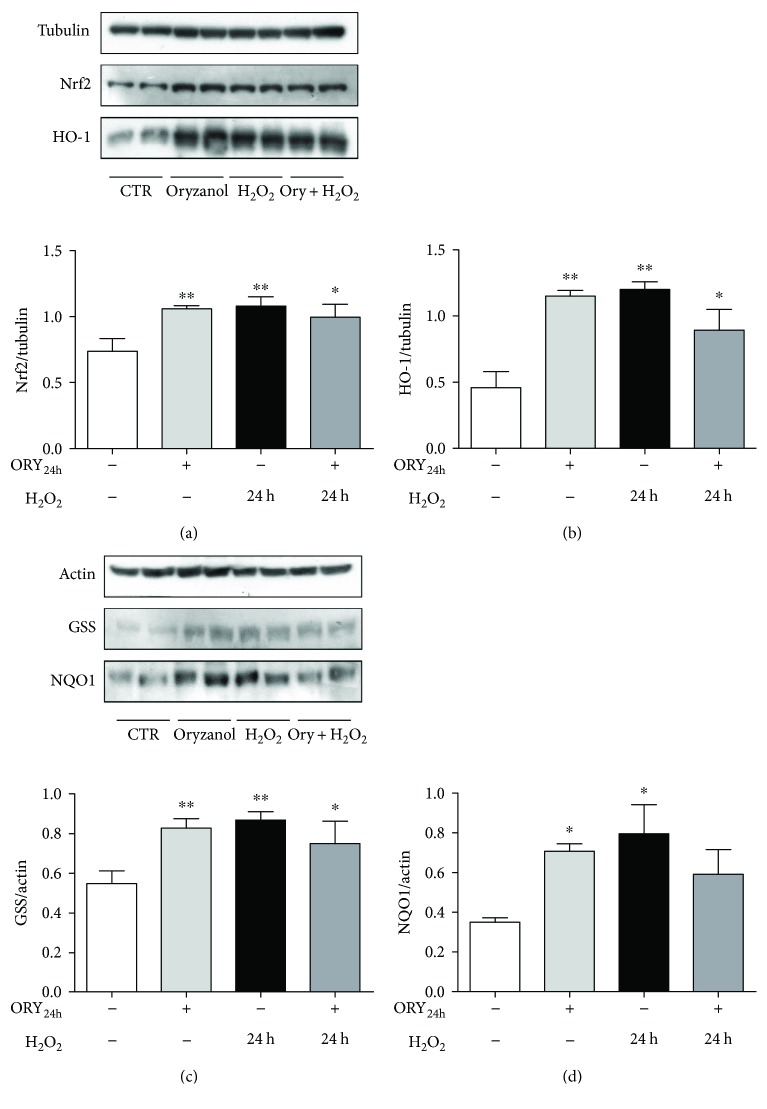
Oryzanol induces an increase in Nrf2 target gene protein levels. HEK-293 cells were pretreated with 5 *μ*g/mL ORY for 24 h followed by 24 h of 100 *μ*M H_2_O_2_. Cells were then processed for measuring Nrf2 (a), HO-1 (b), GSS (c), and NQO1 (d) protein levels by Western blotting. Tubulin and actin were used as loading control according with the molecular weight of the above investigated proteins. Data are shown as mean ± SD; ^∗∗^*p* ≤ 0.01 and ^∗^*p* ≤ 0.05 versus untreated cells, Dunnett's multiple comparison test.

The mRNA expression of Nrf2 was also evaluated in cells pretreated with ORY_24h_ alone or followed by H_2_O_2_ exposure in a time course (Figure 4(a)). H_2_O_2_ alone transiently increased mRNA levels of Nrf2. ORY_24h_ alone did not affect Nrf2 expression. However, when the oxidative insult was present, ORY_24h_ significantly increased its mRNA levels at 1 and 3 h without changing the transient pattern. The increase of mRNA presented also an increase of Nrf2 protein levels at 24 h after H_2_O_2_ in presence or absence of ORY_24h_ (Figure 5(a)).

Since Keap1 is the other key factor involved in the regulation of Nrf2 pathway activation, its mRNA expression was also investigated (Figure 4(b)). ORY_24h_ did not change Keap1 mRNA levels with respect to untreated cells. H_2_O_2_ alone significantly increased Keap1 mRNA level only at 3 h. The combined treatment of ORY_24h_ and H_2_O_2_ although appeared to further enhance Keap1 mRNA, only the results at 3 h were significant.

To verify the complete activation of the Nrf2 pathway, we further analyzed the induction of the cytoprotective Nrf2 target genes. Figures 4 and 5 showed the mRNA expression and protein levels of HO-1 and NQO1 in cells exposed to ORY_24h_ alone or followed by H_2_O_2_.

ORY_24h_ treatment increased the mRNA levels of HO-1 and NQO1 by nearly twofold and onefold, respectively (Figures 4(c)and 4(d)). HO-1 mRNA expression was also transiently induced by H_2_O_2_ with the highest expression at 3 h, which returned to the basal level at 12 h. Interestingly, after treatment with ORY_24h_ followed by the H_2_O_2_, HO-1 mRNA remained high at 12 h (Figure 4(c)). HO-1 protein expression reflected the mRNA results showing a significant increase after ORY_24h_ or H_2_O_2_ alone and remaining high after the combined treatment of ORY_24h_ plus H_2_O_2_ (Figure 5(b)).

Similarly, NQO1 mRNA was transiently increased by H_2_O_2_ alone or in combination with ORY_24h_, while the magnitude of fold increase was lower than that observed for HO-1 at any time points (Figure 4(d)). NQO1 protein expression reflected the mRNA results showing a significant increase after ORY_24h_ or H_2_O_2_ alone and then decreasing after the combined treatment ORY_24h_ plus H_2_O_2_ (Figure 5(d)). Since the increase in GPx activity due to ORY pretreatment, we also decided to investigate protein levels of GSS, another Nrf2 target gene. GSS protein expression was in accordance with the results obtained from HO-1 since it significantly increased after ORY_24h_ or H_2_O_2_ alone and decreased after the combined pretreatment with ORY and H_2_O_2_. These data confirmed an early activation of Nrf2 pathway due to ORY pretreatment highlighting its ability to modulate efficiently the relative expression of Nrf2 target genes at both mRNA and protein levels.

## 4. Discussion

Before the westernization of Eastern countries, the introduction, and the diffusion of Western foods, the Eastern populations were known for their longevity and low incidence of certain illness such as cardiovascular disease [37]. Rice has been at the base of diet of such populations. Moreover, it has been related to the prevention of aging and age-related diseases, but its importance and consumption are not really well claimed. Rice exerts beneficial effects on health as a source of fiber, minerals, vitamins, and phenols with antioxidant activities [19, 20, 38, 39]. Rice-derived ORY has been well studied for its ability to prevent oxidative stress by inducing antioxidant enzyme expression and activity [16, 22].

Our results have showed the antioxidant effects exerted by ORY in an *in vitro* model via inhibition of H_2_O_2_-induced ROS/RNS generation. In particular, we showed that in basal condition, ORY regulated the protein expression and the activity of SOD enzymes. Furthermore, GPx enzyme activity was also enhanced by ORY in H_2_O_2_-induced ROS/RNS and baseline condition. In addition, following the H_2_O_2_ treatment, ORY was able to sustain the antioxidant cellular response and maintain a higher enzymatic activity, thus efficiently turning off ROS/RNS generation. Through this mechanism, ORY might represent a valid first line of defense against oxidative stress. Moreover, beyond its antioxidant properties, ORY was found to possess further potential functions such as antihyperlipidemic, antidiabetic, and anti-inflammatory effects, suggesting a potential role against the development of the related diseases [16, 22, 39–41]. All these disorders are related to redox imbalance, thus suggesting that other mechanisms associated with the regulation of redox homeostasis and the protection against long-lasting oxidative insults could be involved. Here, we showed that ORY exhibited a remarkable free radical scavenging property, and we further deepened the antioxidant profile of ORY by suggesting its ability to trigger the Nrf2 pathway in terms of upregulation of Nrf2 expression, translocation into the nucleus, and induction of the Nrf2-dependent defensive genes such as HO-1, NQO1, and GSS. These results are very interesting because they might promote ORY or ORY-inspired compounds as an integrative intervention to impact the aging process and longevity together with the decrease of oxidative stress [42, 43].

**Figure 6:**
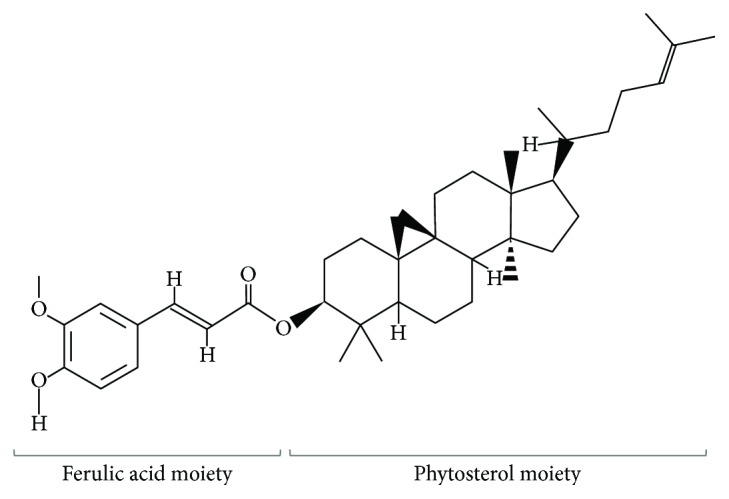
Molecular structure of oryzanol. The structure of ORY consists of two moieties: ferulic acid and phytosterols (National Center for Biotechnology Information; PubChem Compound Database, CID = 6450219.)

Induction by oxidative stress is the best-understood mechanism of Nrf2 activation. In our experimental model, H_2_O_2_ was able to induce Nrf2 translocation and activation of its target genes involved in cellular responses against xenobiotics. NQO, HO-1 mRNA, and protein levels were found enhanced in HEK-293 cells treated with H_2_O_2_. SOD1 is another target gene of Nrf2. H_2_O_2_ induced an increase in the expression of SOD1 suggesting that it could be under the regulation of Nrf2. The mechanism by which free radicals activated Nrf2 pathway is via the modification of cysteine residues on Keap1, thus resulting in Nrf2 accumulation in the cytoplasm and translocation into the nucleus. The oxidative insult also induced the increase of mRNA and protein expression of Nrf2 (Figure 4(a)). Two ARE-like motifs in the 5 flanking regions of the Nrf2 promoter are responsible for the induction of Nrf2 upon activation [44] ensuring a feed-forward process with Nrf2 activation promoting its own expression, thus facilitating a profound cellular response to stress. Therefore, all these data highlighted that the cells were unable to detoxify themselves from the oxidative stress induced by ROS/RNS production. In addition, Nrf2 can also be activated by different phytochemicals [45–47] as well as various pharmaceuticals (reviewed in [48]) via overlapping and distinct mechanisms. Here, we demonstrated that ORY alone was able to transiently induce Nrf2 translocation that was still present when oxidative insult was added. Our hypothesis is that the mechanism by which ORY is able to modulate Nrf2 pathway might be due to its intrinsic chemical structure (Figure 6) [49]. The presence of ROS/RNS induces the hydroxyl group on the phenolic ring of ORY [16, 22] resulting in the formation of a phenoxy radical, which might be involved in the modification of cysteine thiols of Keap1 leading to the inhibition of Keap1-Nrf2 binding. As a consequence of Nrf2 nuclear translocation, its target genes are transactivated. Here, we found that ORY_24h_ induced the transient transcription of HO-1, NQO1, and GSS genes by the increase of their protein expression. It is noteworthy that HO-1 mRNA was still higher after 12 h of pretreatment with ORY followed by H_2_O_2_ exposure suggesting the presence of alternative mechanisms to sustain in parallel its expression. Finally, after ORY_24h_ pretreatment, the increase of SOD1 protein expression could be also influenced by the Nrf2 pathway.

In conclusion, this study suggested that the antioxidant effects of ORY could also be sustained by the Nrf2 pathway. Nrf2 is involved in regulating longevity and age-related diseases and has also been proposed as a master regulator of the aging process. Further *in vitro* and *in vivo* studies of ORY as a potential longevity-promoting inducer could be studied in the future.
